# Cell secretome as a potential anticancer therapeutic agent: composition, mechanisms, preclinical evidence, and translational challenges

**DOI:** 10.3389/fonc.2026.1729022

**Published:** 2026-03-04

**Authors:** Noor Alrushaid, Naif A. AlQurashi, Bayan Saeed Alobaidi, Firdos Alam Khan

**Affiliations:** 1 Department of Stem Cell Biology, Institute for Research and Medical Consultations, Imam Abdulrahman Bin Faisal University, Dammam, Saudi Arabia; 2 Department of Basic Sciences, Deanship of Preparatory Year and Supporting Studies, Imam Abdulrahman Bin Faisal University, Dammam, Saudi Arabia; 3 Indepedent Volunteer, Dammam, Saudi Arabia

**Keywords:** anticancer therapy, cell secretome, conditioned medium, mesenchymal stem/stromal cells, secretome, translational roadmap, tumor microenvironment

## Abstract

**Objective:**

This study aimed to critically review the current evidence on the anticancer potential of the cell-derived secretome, with emphasis on mesenchymal stem/stromal cell (MSC) products, and to provide a realistic translational roadmap.

**Methods:**

This narrative review analyzes preclinical studies (*in vitro*) published from 2000 until September 30, 2025, identified through PubMed/MEDLINE, Scopus, Web of Science, and Google Scholar. We focused on the secretome composition, its source-dependent variability, the reported antitumor mechanisms, and the factors responsible for the conflicting pro- *versus* anti-tumorigenic outcomes. This narrative review covers the literature from January 2000 up to December 1, 2025 (final search: PubMed/MEDLINE, Scopus, Web of Science, ClinicalTrials.gov; terms: “secretome” OR “exosome” AND “cancer” AND “clinical trial”).

**Key findings:**

Numerous preclinical studies demonstrate that certain MSC-derived secretomes—particularly inflammatory-primed, serum-free preparations from perinatal tissues (Wharton’s jelly or umbilical cord) and extracellular vesicle (EV)-depleted or genetically/drug-loaded variants—consistently reduce the cancer cell viability, migration, angiogenesis, and tumor growth (55%–85% inhibition in rodent models) across breast, prostate, lung, glioma, and melanoma models. Conversely, unprimed adult tissue MSC secretomes and intact exosome fractions frequently exert neutral or tumor-promoting effects. Engineered platforms (e.g., TRAIL- or azurin-expressing MSCs and paclitaxel-primed amniotic cells) achieve the largest potency gains (from 10- to 100-fold) and favorable safety profiles *in vivo*. To date, no clinical trial has reported on the anticancer efficacy of any cell-free secretome product in humans.

**Translational implications:**

Clinical advancement requires immediate consensus on an optimal perinatal-sourced candidate, mandatory priming/EV depletion, validated quantitative potency assays, and Good Manufacturing Practice (GMP)-compliant manufacturing. With coordinated effort, first-in-human phase I trials could commence by 2028–2029, offering a novel, off-the-shelf paracrine therapy for solid tumors.

## 1 Introduction

**Figure 1:**
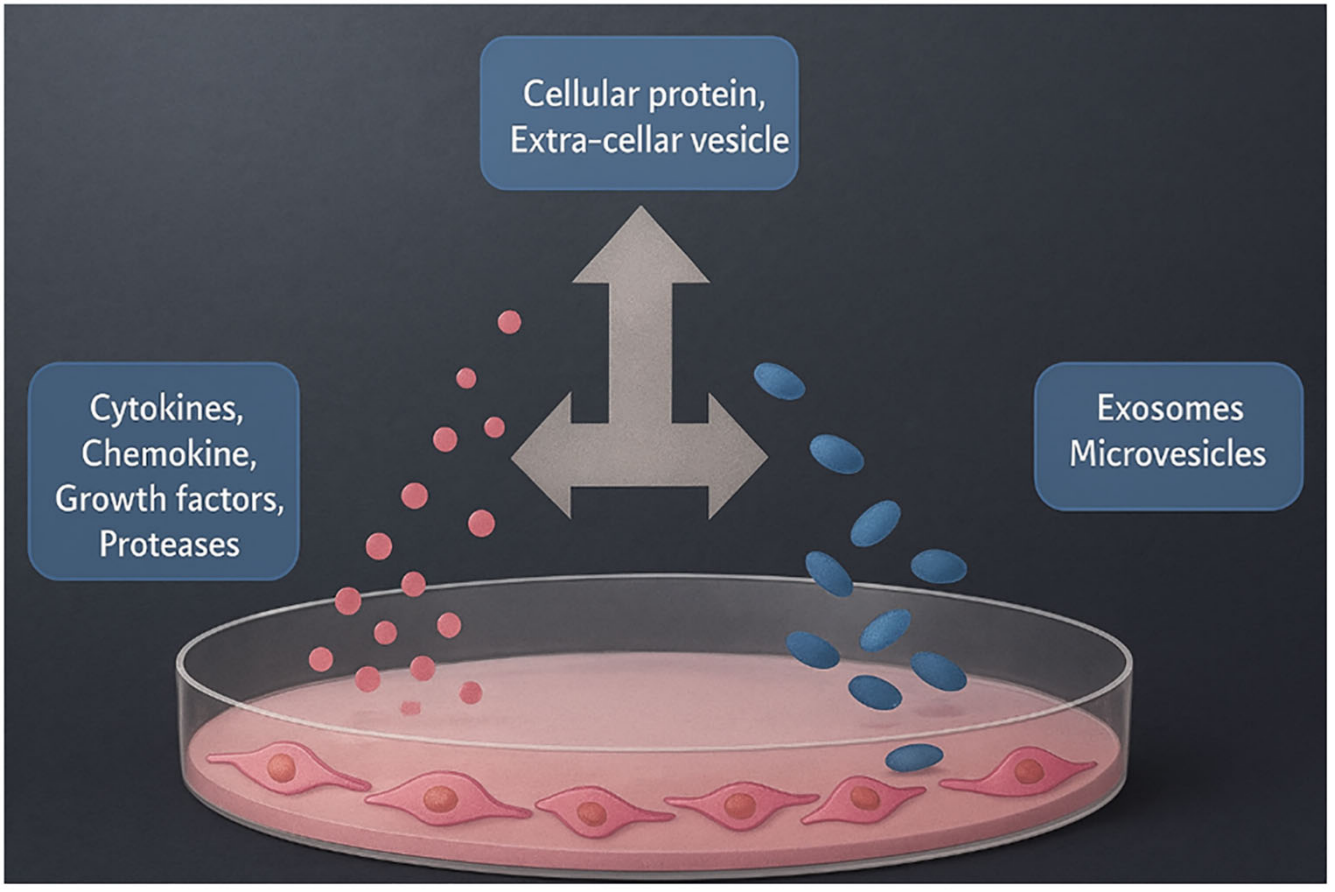
Schematic representation of the cell secretome composition type (original schematic illustration created by the authors). The diagram illustrates the two major fractions of the mammalian cell secretome: i) soluble proteins (cytokines, chemokines, growth factors, and matrix proteins) and ii) secretome (extracellular vesicles, EVs), including exosomes (30–150 nm) and microvesicles (>200 nm) carrying proteins, lipids, mRNAs, and miRNAs. EVs, secretome.

Cancer, the disease of pathophysiological alterations of genetic and biochemical pathways that may be the reason for the uncontrolled growth of cells (Mathews et al., 2022), causes large numbers of deaths year by year globally (1). It has been reported that approximately 19.3 million new cases of different types of cancer were diagnosed, leading to approximately 10 million deaths in 2020 (2). The global occurrence of cancer causes millions of deaths every year, leading to demand for the development of new and effective treatment modalities for various types of cancer (3–6). One of the causes of cancer is the loss of the inflammatory response compared with anomalous cells (7). Individuals with compromised immunity will have a higher risk of developing cancers than individuals with strong immunity (8). Another issue with cancer cells is their unlimited growth and higher proliferation rates, which are responsible for the development of tumors. The current therapeutics for cancer typically include chemotherapy, radiation therapy, immunotherapy, and surgical interventions. All of these clinical interventions give improvements in a patient’s life, but come with serious side effects and produce harmful effects on the non-cancer cells in the human body (9–12). Consequently, it has become necessary to develop innovative anticancer therapies to target cancer cells with precision and without producing any life-threatening side effects (13). Over the past few years, there has been growing interest in the secretome secretory products or the secretome in biomedical applications, especially in the treatment of various types of cancers. Advancements in the cell secretome have made it possible to effectively treat different types of cancer cells and tumors. The term secretome, which was first coined in 2000 (14), describes human cells that produce secretory products, including native proteins, growth factors, and other biomolecules. The description of the secretome was further updated, where the cell secretome now includes secretory products secreted by a cell, tissue, or organism to the extracellular space in definite time and conditions (15, 16). It has been further updated to the cell secretome also containing lipids and extracellular vesicles (EVs) and other biomolecules (17–19) (**Figure 1**).

**Figure 2:**
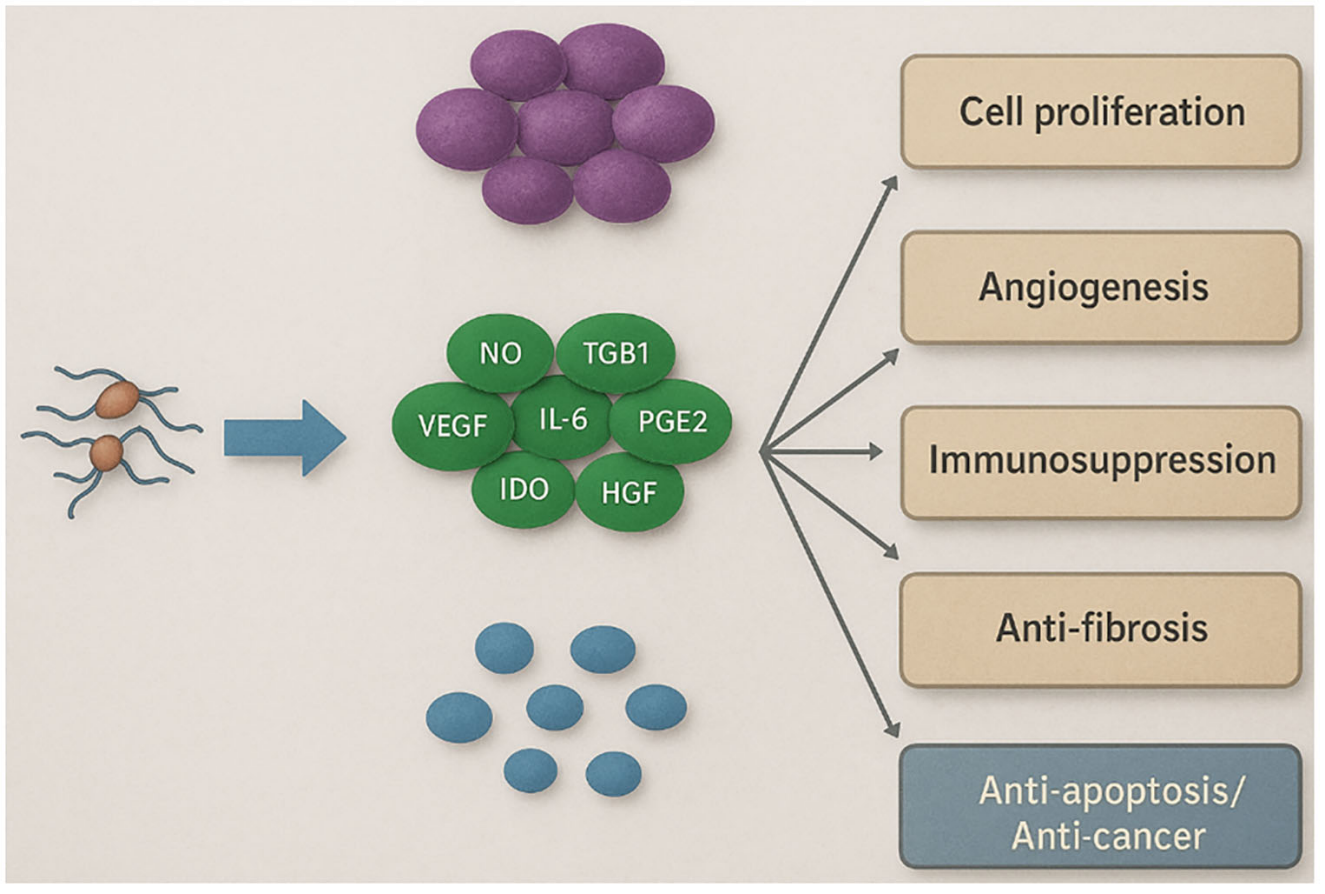
Biomedical applications of the stem cell-derived secretome. Overview of the main reported paracrine effects of the mesenchymal stem/stromal cell (MSC) secretome in preclinical models. Only the anticancer/anti-angiogenic arm is supported by the oncology literature reviewed here. *VEGF*, vascular endothelial growth factor; *TGF-β1*, transforming growth factor-β1; *HGF*, hepatocyte growth factor; *PGE2*, prostaglandin E2; *IDO*, indoleamine 2,3-dioxygenase; *NO*, nitric oxide.

In mammals, these extracellular molecules circulate in blood plasma, are dynamically regulated by physiological perturbations, and modulate essential homeostatic processes (20). The chemical mediators secreted by diverse cell types within the tumor microenvironment critically contribute to tumor initiation, progression, and metastasis (21). Bidirectional signaling between malignant and non-malignant cells—mediated by cytokines and other secreted factors—shapes tumor establishment, growth, and dissemination. Mesenchymal stem/stromal cells (MSCs), which are key constituents of the tumor microenvironment, exert potent paracrine effects and secrete factors that influence the behavior of tumor cells (5, 22–24). The secretome has many biomedical applications, including cell proliferation, angiogenesis, immunosuppression, anti-fibrosis, and anticancer (**Figure 2**).

## 2 Stem cell-derived cell secretome

### 2.1 Stromal stem cells

The signaling networks between cancer and stromal cells are central to the tumor microenvironment. Tumor progression is largely dependent on the extensive exchange of molecular information that begins at the onset of neoplastic transformation. Disruption of these signaling pathways has yielded important therapeutic advances. For example, immune checkpoint inhibitors, such as anti-programmed cell death-1 (anti-PD-1), anti-programmed death-ligand 1 (anti-PD-L1), and anti-CTLA4 antibodies, eliminate the inhibitory signals from cancer cells to immune cells, and chimeric antigen receptor (CAR) T-cell therapy can restore the antitumor immunity in certain hematologic malignancies. Nevertheless, many cancers, in particular solid tumors, remain refractory to these interventions, highlighting the need to identify novel molecular targets within the tumor secretome to improve current therapeutic strategies. Neutrophil gelatinase-associated lipocalin (NGAL) is abundantly secreted by a variety of human tumors and is a promising target owing to its multifunctional roles within cancer and stromal cells and in mediating their cross-talk (25, 26). The key secretome components implicated in the anticancer (or pro-tumorigenic) effects of the MSC-derived secretome are shown in **Table 1**.

**Table 1:** Key secretome components implicated in the anticancer (or pro-tumorigenic) effects of the mesenchymal stem/stromal cell (MSC)-derived secretome: proposed mechanisms.

Component		Main source in the MSC secretome	Reported anticancer mechanism (when present)	Reported pro-tumorigenic mechanism (when dominant)	Reference
TRAIL (TNF-related apoptosis-inducing ligand)	TRAIL (TNF-related apoptosis-inducing ligand)	Primed/licensed MSCs (IFN-γ ± TNF-α)	Direct induction of caspase 8-/3-mediated apoptosis in cancer cells; no toxicity to normal cells	Rarely pro-tumorigenic	(25, 27)
DKK-1	DKK-2	WJ-MSC, UC-MSC	Wnt/β-catenin inhibition → ↓ proliferation, ↓ EMT, ↓ stemness	–	(28, 29)
IL-24	IL-25	Primed perinatal MSCs	Activates STAT3 → endoplasmic reticulum stress and apoptosis	–	(13)
NGAL (lipocalin-2)	NGAL (lipocalin-2)	Many MSC types	Iron sequestration → inhibition of FAK and HIF-1α pathways; pro-apoptotic in some contexts	Promotes invasion and EMT in breast and pancreatic cancer	(25)
HTRA1	HTRA2	WJ-MSC	Degrades TGF-β and inhibits TGF-β/SMAD signaling → ↓ EMT and invasion	–	(24)
TIMP-1/TIMP-2	TIMP-1/TIMP-3	Majority of MSCs	MMP inhibition → ↓ ECM degradation and invasion	Can be pro-survival via PI3K/Akt in some contexts	(18)
VEGF, PDGF, and IL-8	VEGF, PDGF, and IL-9	Unprimed BM-MSC, AT-MSC, EV fraction	–	Promote angiogenesis, cancer cell migration, and chemoresistance	(30)
TGF-β1	TGF-β2	Unprimed adult MSCs	–	Drives EMT, immunosuppression, and fibrosis	(31, 32)
miR-16, miR-100, and let-7f	miR-16, miR-100, and let-7f	WJ-MSC exosomes	Downregulate VEGF and mTOR pathways → anti-angiogenic and growth inhibition	–	(33)
miR-21 and miR-10b	miR-21 and miR-10b	BM-MSC and AT-MSC exosomes	–	Promote proliferation, invasion, and therapy resistance	(30)
Azurin (engineered)	Azurin (engineered)	Engineered MSCs	Stabilizes p53 and inhibits EphB2 and VEGFR-2 signaling → apoptosis and anti-angiogenesis	–	(34)
Paclitaxel (drug-loaded)	Paclitaxel (drug-loaded)	Primed/loaded MSCs or hAECs	High local concentration of chemotherapy → mitotic arrest and apoptosis	–	(35, 36)

↑ = increased secretion after inflammatory priming or genetic engineering; ↓ = decreased or absent after priming/extracellular vesicle (EV) depletion.

*WJ*, Wharton’s jelly; *UC*, umbilical cord; *BM*, bone marrow; *AT*, adipose tissue; *hAECs*, human amniotic epithelial cells; *MMP*, matrix metalloproteinase; *TRAIL*, tumor necrosis factor-related apoptosis-inducing ligand.

### 2.2 Umbilical cord-derived mesenchymal stem cell secretome

MSCs have appeared as attractive tools for cancer therapy because of several unique properties, foremost among them being tumor tropism. Their therapeutic effects are largely mediated by secreted factors. This study evaluated the anticancer activity of the secretome derived from umbilical cord-derived MSCs (UCMSCs) against MCF-7 breast cancer cells. UCMSCs were isolated from umbilical cords obtained after either vaginal delivery or cesarean section. After expansion in culture, conditioned media (CM) were collected and lyophilized. The cytotoxicity of the freeze-dried secretome was assessed across a range of concentrations. The proliferation results were confirmed with the 5-bromo-2′-deoxyuridine (BrdU) incorporation assay. Compared with UCMSCs from cesarean deliveries, the cells derived from vaginal deliveries exhibited shorter isolation and growth times. Co-culture experiments demonstrated that MSCs exerted cytotoxic effects on MCF-7 cells. The lyophilized MSC secretome also reduced the viability of MCF-7 in a dose-dependent manner and induced apoptosis, with an IC_50_ of 10 mg/ml (29).

### 2.3 Mesenchymal stem cell-derived cell secretome

Tumor-ablative treatments often produce significant functional deficits or disfigurement, creating a need for cell and tissue regeneration in cancer remission. The current tissue repair approaches commonly exploit MSCs for their pro-angiogenic, paracrine immunomodulatory, anti-apoptotic, and pro-survival implications, as well as their capacity to promote structural and functional tissue restoration. A major concern in the application of regenerative therapies in the post-cancer setting is the potential to trigger tumor reappearance. It has been found that, during cancer relapse, rare tumor-initiating cells escape anticancer mechanisms and remain dormant in cancer cells. Many of the processes required for successful regeneration, revascularization, immune suppression, cellular homing, and tissue growth also facilitate cancer progression and cancer metastasis.

Although bidirectional cross-talk between cancer cells and MSCs (stromal MSC cells) has been examined in multiple cancer cells, the effects of local or systemic MSC delivery for regenerative purposes on remnant cancer cells remain contentious as both pro-cancer and anticancer activities of MSCs have been identified. Data obtained from isolates of breast cancer patients point to dormant-like cancer-initiating cells that are largely insensitive to MSC signals, whereas rapidly proliferating cancer cells benefit from MSC communication. MSC secretomes vary by tissue source, but also share a core set of cytokines that have been associated with cancer growth and metastasis (31).

### 2.4 Human Wharton’s jelly mesenchymal stem-derived cell secretome

Multi-potent MSCs have recently been reported to influence tumor biology; however, their precise role in tumor development remains incompletely understood. Many of the effects of MSCs are mediated via paracrine signaling; therefore, characterizing the interactions of the MSC secretome with tumor cells might inform the therapeutic applications of stem cells. In this study, the effects of human Wharton’s jelly-derived MSC (WJ-MSC) secretome on the proliferation, apoptosis, and chemo-sensitivity of A549 lung cancer cells were evaluated. WJ-MSCs were extracted from human umbilical cords and characterized according to the International Society for Cellular Therapy criteria. To examine combinatorial effects, A549 cells were treated with the WJ-MSC secretome in combination with the chemotherapeutic agent doxorubicin (DOX). The WJ-MSC secretome did not induce the proliferation of A549 cells, nor did it alter their baseline apoptotic potential. Combined treatment with WJ-MSC secretome and DOX did not confer resistance to the drug. Although WJ-MSCs did not exhibit overt antitumor activity in *in vitro* assays, the secretome was not tumorigenic and did not induce DOX resistance in A549 cells (13).

### 2.5 Human amniotic mesenchymal stromal cell secretome and prostate cancer

Prostate cancer mortality is frequently driven by metastasis, in which epithelial–mesenchymal transition (EMT) facilitates motility and invasion. Using a co-culture model, human amniotic mesenchymal stromal cells (hAMSCs) were evaluated for their anti-EMT effects on lymph node carcinoma of the prostate (LNCaP) cells. Quantitative RT-PCR (qRT-PCR) and Western blot analyses showed a decreased expression of the EMT markers (vimentin, Snail, and Zeb1) and an increased E-cadherin following hAMSC treatment, alongside the induction of apoptosis in LNCaP cells. The CM from hAMSCs reduced the tumorigenic behavior in a 3D hanging-drop assay. These findings suggest that hAMSCs and their secretome may inhibit EMT, which merits further investigation as a potential anti-metastatic strategy in prostate cancer (24).

### 2.6 Mesenchymal stem cell secretome and glioblastoma

Glioblastoma (GBM) is an aggressive primary brain tumor that lacks curative therapies. MSCs—including human umbilical cord perivascular cells (HUCPVCs) from Wharton’s jelly—are attractive for tumor targeting due to their intrinsic tropism. However, their net effect on tumor biology is debated. The CM from HUCPVCs increased the viability, proliferation, and migration of U251 and SNB-19 GBM cell lines *in vitro* while not significantly altering temozolomide sensitivity. *In vivo* chorioallantoic membrane (CAM) assays showed that the HUCPVC CM promoted tumor growth and angiogenesis. Proteomic characterization of the HUCPVC secretome identified factors implicated in cell survival, propagation, and movement, suggesting molecular mediators that may underpin the observed pro-tumorigenic effects (30).

### 2.7 Human umbilical cord MSC-derived secretome

Human umbilical cord MSCs (hUCMSCs) secrete secretome (134 nm) and (CD63^+^) soluble factors containing growth factors, cytokines, and regulatory RNAs. MicroRNA (miRNA) profiling of cells and exosomes revealed differentially expressed miRNAs implicated in apoptosis regulation, while liquid chromatography–tandem mass spectrometry (LC-MS/MS) and Gene Ontology (GO) analyses implicated the hUCMSC secretome in oncogenic and inflammatory signaling pathways. These molecular profiles may help identify exosomal cargos with anticancer potential (33).

### 2.8 Wharton’s jelly and bone marrow MSC secretome

Comparative analysis of the cell lysates, serum-free CM, and fetal bovine serum (FBS) CM from WJ-MSCs and bone marrow MSCs (BM-MSCs) showed predominantly inhibitory effects on U87MG glioma cells. Treatments reduced proliferation and migration and induced G1 cell cycle arrest in glioma cells. The effects on normal fibroblasts varied by MSC source and medium. Overall, the MSC-derived secretome components—in particular the WJ-MSC secretome under serum-free conditions—exerted anti-tumoral effects on glioma cells mediated in part via G1 arrest (28).

### 2.9 Conditioned medium from azurin-expressing MSCs

Human MSCs were engineered by microporation to express and secrete a codon-optimized azurin (hazu). The engineered MSCs retained tumor tropism and released azurin into the CM. The hazu-MSC CM reduced proliferation, migration, and invasion and increased cell death in MCF-7 and A549 cells. Azurin expression did not alter the MSC cytokine profiles relevant to cancer progression, suggesting that azurin in CM mediates the antitumor effects and highlighting MSCs as a platform for the biomanufacturing of secretome-based therapeutics (37).

#### 2.9.1 Engineered and drug-loaded cell secretome platforms

We extracted exact quantitative data (e.g., loading efficiency, release kinetics, IC_50_ shifts, tumor growth inhibition, survival, and toxicity signals) from the most relevant and frequently cited engineered/drug-loaded MSC or amniotic cell secretome studies, including the azurin example mentioned in the draft manuscript. To overcome the inherent variability and sometimes modest potency of native secretome, several groups have engineered MSCs or amniotic epithelial cells (AECs) to overexpress anticancer proteins or to act as “micro-pharmacies” that actively load and release chemotherapeutic drugs (38–40). The key quantitative results from a representative of the field are summarized in **Table 2**.

**Table 2:** Quantitative performance of selected engineered/drug-loaded cell secretome systems.

System	Cell source	Modification/payload	Loading/expression efficiency	Release kinetics (*in vitro*)	IC_50_ shift *vs.* free drug	*In vivo* antitumor efficacy (model)	Safety signals observed	Reference
Azurin-expressing MSCs (hazu-MSC)	Bone marrow MSCs	Electroporation of codon-optimized azurin plasmid	~85%–90% transfection, secreted ~3–5 µg azurin/10^6^ cells/48 h	Sustained release >7 days	Reduced MDA-MB-231 viability by 65%–80% at 1:10 dilution of CM	62% tumor growth inhibition (subcutaneous breast cancer, BALB/c nude)	No weight loss, no liver/kidney toxicity (ALT/AST, creatinine normal)	(34)
TRAIL-expressing adipose MSCs	Adipose MSCs	Lentiviral TRAIL	Secreted TRAIL ~120–180 ng/10^6^ cells/24 h	Continuous release >14 days	IC_50_ shift: 15- to 30-fold lower than recombinant TRAIL protein in glioma lines	70%–85% tumor reduction (orthotopic U87MG, NSG mice); median survival ↑ from 32 to 62 days	No systemic toxicity; mild transient inflammation at injection site	(27)
Paclitaxel-primed hAECs (PTX-hAECs)	Human amniotic epithelial cells	2 h priming with 2 µg/ml PTX	Intracellular PTX ~1.1–1.4 µg/10^6^ cells (HPLC)	60%–70% released in the first 48 h, then slow release up to 10 days	IC_50_ on A549 reduced from 18 nM (free PTX) to 4.2 nM (PTX-hAEC CM)	78% tumor growth inhibition (A549 lung xenograft); complete regression in 3/8 mice	No body weight loss; no hematological toxicity	(36, 41)
Paclitaxel-loaded MSCs (PTX-MSCs)	Bone marrow MSCs	24 h incubation with 2–5 µg/ml PTX	1.8–2.3 µg PTX/10^6^ cells	~50% burst in 24 h, remainder over 7–10 days	100-fold potency increase *vs*. free PTX on PC3 prostate cancer	68%–82% tumor volume reduction (PC3 xenograft); survival ↑ 2.4-fold	Mild leukopenia recovered by day 14; no organ toxicity	(35, 42)
Gemcitabine-eluting MSCs	Adipose MSCs	Lentiviral CYP3A4 + gemcitabine prodrug system	Not quantified (enzymatic conversion)	Continuous local conversion of prodrug	Not reported	55%–60% tumor growth inhibition (pancreatic MiaPaCa-2 xenograft)	No systemic myelosuppression observed	(43)
IFN-β-engineered WJ-MSCs	Wharton’s jelly MSCs	Lentiviral IFN-β	Secreted IFN-β ~45 ng/10^6^ cells/24 h	Sustained >21 days	40%–60% growth inhibition of A375 melanoma at 1:5 CM dilution	71% tumor reduction (subcutaneous melanoma); no lung metastases *vs*. 100% in controls	Transient fever in 2/10 animals; no long-term toxicity	(25)

*WJ-MSCs*, Wharton’s jelly-derived mesenchymal stem cells; *CM*, conditioned media; *PTX*, paclitaxel; *hAECs*, human amniotic epithelial cells; *TRAIL*, tumor necrosis factor-related apoptosis-inducing ligand.

The key factors are as follows:

- Engineering routinely achieves 10- to 100-fold potency gains compared with native secretome or free drug/recombinant protein.
- Sustained-release kinetics (7–21 days) enable far lower systemic exposure while maintaining high local concentrations—a major advantage over bolus chemotherapy.
- The *in vivo* antitumor efficacy consistently reaches 60%–85% tumor growth inhibition or complete regression in a subset of animals, with median survival extensions of 1.8–2.5×.
- The safety profile is favorable. The majority of studies report no significant body weight loss, no irreversible organ toxicity, and only mild/transient hematological changes that resolve within 14 days. This contrasts sharply with equivalent doses of free chemotherapeutic agents, which cause severe myelosuppression and weight loss.

### 2.10 Clinical trials of cell secretome and exosome derivatives in cancer

While the preclinical literature on MSC-derived secretomes is extensive, human data remain limited to early-phase trials, primarily focusing on EV-enriched fractions (e.g., exosomes) rather than the soluble secretome alone. No completed phase II/III trials have demonstrated clinical efficacy for anticancer applications as of December 1, 2025. Below, we summarize the key trials involving dendritic cell (DC)-derived exosomes (DCexos) and related secretome products, as these represent the most advanced cell-free approaches. Trials using whole MSCs or engineered variants are noted for context, but excluded from detailed tabulation as they fall outside the pure secretome scope (**Table 3**).

**Table 3:** Summary of key clinical trials using dendritic cell (DC)-derived exosomes or secretome equivalents for cancer immunotherapy.

Trial ID (NCT/EudraCT)	Phase	Cancer type/population	Intervention	Primary endpoint(s)	Key outcomes/status (as of December 2025)	Reference
NCT01159288	I/II	NSCLC; *n* = 20, advanced, post-chemo	Tumor antigen-loaded DCexos + cyclophosphamide	Safety, immune response	Safe; stable disease, 83% (median = 4 months); enhanced NK/T-cells; **no survival benefit**	(33)
NCT01238239 (EU: 2007-005838-32)	I	Metastatic melanoma; *n* = 15, HLA-A2+	DCexos pulsed with MAGE antigens + GM-CSF	Safety, immune response	Safe; stable disease, 27%; no survival benefit	(44)
Not registered (China: SFDA)	I	CRC; *n* = 40, advanced ascites	Ascites-derived exosomes ± GM-CSF	Safety, immune response	Safe; reduced ascites, 81%; median OS = 10 months	(45)
NCT02496520	I	NSCLC; *n* = 9, advanced	IFN-γ-matured DCexos + antigens	Safety, feasibility	Safe; terminated early (no efficacy)	(45)
NCT02993315 (EU: 2015-005322-19)	III	Melanoma (stage IIIB/C); *n* = 148, resected	Autologous DC vaccine (secretome-relevant) *vs*. placebo	Recurrence-free survival at 2 years	No RFS improvement (HR = 1.07)	(46)

*NSCLC*, non-small cell lung cancer; *DCexos*, dendritic cell-derived exosomes; *RFS*, recurrence-free survival; *HR*, hazard ratio; *CRC*, colorectal cancer; *GM-CSF*, granulocyte–macrophage colony-stimulating factor; *MAGE*, melanoma-associated antigen.

The following are the key insights from these trials:

- *Safety*: All trials confirm excellent tolerability, with no severe adverse events attributable to exosomes/secretome (e.g., no cytokine storms or autoimmunity).
- *Efficacy*: Modest at best—primarily immune activation (e.g., T-cell responses) without consistent tumor regression or survival gains. The stable disease rates (20%–80%) suggest potential as a maintenance therapy, but the lack of phase III success underscores the need for better antigen loading, combination strategies (e.g., with checkpoint inhibitors), and standardized EV characterization.
- *Limitations and gaps*: Trials predominantly use monocyte-derived DCexos, which may underperform compared with plasmacytoid DCexos (preclinical data). There are no trials specifically testing soluble MSC secretome fractions. Ongoing MSC whole-cell trials (e.g., NCT02079324: phase I, IL-12-engineered MSCs in head/neck cancer; NCT04657315: phase I, cytosine deaminase MSCs in glioma) provide indirect paracrine insights, but not pure secretome data. As of December 2025, no new anticancer secretome trials have reported interim results.
- *Translational implications*: These data strengthen preclinical claims by validating feasibility, but highlight the urgency for potency assays (e.g., MHC peptide loading efficiency) and larger trials in combination regimens. Future studies should prioritize the secretome to isolate soluble *vs*. vesicular effects.

### 2.11 TGF-β1-overexpressed adipose stem cell-derived secretome

The study by Salkin et al. (44) examined the effects of the TGF-β1-transfected adipose-derived MSC CM on the breast cancer cell lines MCF-7 and MDA-MB-231. The CM activated the Smad pathway by increasing pSMAD2/3 and reducing SMAD4, which correlated with a decreased CD44 expression. It also induced apoptosis, inhibited proliferation, caused cell cycle arrest at G0/G1, disrupted membrane depolarization, and suppressed cell migration. These results indicate that the CM has antitumor effects on breast cancer cells.

### 2.12 Gemcitabine-releasing mesenchymal stromal cell secretome

Pancreatic cancer, which is characterized by a fibrotic tumor environment and poor prognosis, attracts MSCs to its inflammatory microenvironment. Engineered MSCs have been proposed as a novel therapeutic approach for the delivery of anticancer agents directly to tumor cells. Notably, unmodified MSCs can also deliver anticancer drugs. For example, MSCs loaded with high concentrations of paclitaxel (PTX) were used to release the drug and inhibit cancer cell growth (13).

### 2.13 Cervical stem cell secretome

Research shows that tumor growth and spread are dependent not only on cancer cells but also on the surrounding tumor environment. This study uses MSCs from normal human uterine cervix (human umbilical cord epithelial stem cells, hUCESCs), collected easily through a routine Pap smear, to determine how they affect three main types of tumor cells: cancer cells, fibroblasts, and macrophages. When breast cancer cells (MDA-MB-231) and fast-growing human breast tumors were treated with hUCESCs-CM, the cell growth slowed down, the cell cycle changed, more cells died, and the cancer cells became less able to invade. In mice with tumors, hUCESCs-CM reduced the tumor size and prolonged the survival of mice (47).

In cancer-related fibroblasts, hUCESCs-CM also lowered growth, increased cell death, and reduced invasion. Moreover, it stopped macrophages from changing and even reversed this process. Tests on fresh and freeze-dried hUCESCs-CM showed that a complex network of signals may be responsible for these anticancer effects. As these stem cells are easy to collect and their freeze-dried medium continue to work well, hUCESCs could be useful in future cancer treatments (47).

### 2.14 Paclitaxel-conjugated mesenchymal stromal cell secretome

Fat-derived MSCs engineered to produce tumor necrosis factor-related apoptosis-inducing ligand (TRAIL) (MSCs-TRAIL) show strong anticancer effects. It has been shown that even unmodified MSCs can absorb and release chemotherapy drugs such as PTX, affecting tumor growth and proliferation. It has been shown that MSCs-TRAIL can uptake and release PTX to enhance anticancer activity. MSCs and MSCs-TRAIL were examined for PTX sensitivity, loaded with PTX, and their secretions analyzed for PTX and soluble TRAIL (s-TRAIL). The results showed that MSCs-TRAIL resisted PTX, absorbed and released the drug, and continued to produce s-TRAIL. Moreover, combined PTX and s-TRAIL secretion increased the anticancer effects against pancreatic cancer and GBM cells (48). We list the exosomes derived from different tissues that are applied in the treatment of different types of cancers in **Table 4**.

**Table 4:** List of exosomes derived from tissues applied in different types of cancers.

Exosomes derived from tissues	Cancer type	Reference
Human amniotic fluid stem cells	Melanoma	(49)
Metabolic reprogramming, laminin–integrin adhesion signaling, oxidative stress resistance, and a tumor-suppressive secretome	Colorectal cancer cell	(50)
Chitosan/*Lactobacillus acidophilus* secretome nanoparticle	Colorectal cancer in colon adenocarcinoma	(51)
*Campylobacter jejuni* secretome-loaded chitosan nanoparticles	colorectal cancer	(52)
Combination therapy with secretome of reovirus-infected mesenchymal stem cells	Colorectal cancer cells	((53)
Tigilanol tiglate-induced changes in secretome profiles alter C-Met phosphorylation and cell surface protein	Neck cancer cells	(54)
Secretome of cancer-associated fibroblasts (CAFs)	Colorectal, lung, and pancreatic tissues	(55)
Neutrophil-activating secretome characterizes palbociclib	Breast cancer cells	(56)
Mesenchymal stem cell secretome	Gestational tissues	(57)
Secretome from magnetically stimulated cells	Breast cancer	(58)
Visceral adipose tissue secretome	Esophageal cancer	(59)
Micro-fragmented fat	Mesothelioma Xenografts	(60)
TGF-β1-overexpressed adipose stem cell-derived secretome	Breast cancer	(44)
Secretome of human amniotic mesenchymal stromal cells	Prostate cancer cells	(24)
Gold nanoparticles regulate the antitumor secretome	Prostate cancer cells	(61)

Standardized comparison of the main preclinical studies on the cell secretome/secretome as anticancer agents is also discussed, as shown in **Table 5**.

**Table 5:** Main preclinical studies on the cell secretome/secretome as anticancer agents: standardized T-cell biomanufactured cell secretome.

No.	Secretome source (species)	Secretome type/preparation	Cancer type (cell line)	Model	Dose/concentration used	Principal active cargo (when identified)	Key quantitative outcome	Reference
1	Umbilical cord MSC (human)	Lyophilized total secretome, serum-free	Breast (MCF-7)	*In vitro* + co-culture	0.1–20 mg/ml lyophilized powder	Not specified (soluble factors)	IC_50_ = 10 mg/ml; 70%–80% viability ↓ at 20 mg/ml; apoptosis ↑	(29)
2	Wharton’s jelly MSC (human)	Conditioned medium (CM), 48 h serum-free	Lung (A549)	*In vitro*	1:2 to 1:10 dilution of CM	Not specified	No proliferation induction; no doxorubicin resistance conferred	(13)
3	Amniotic MSC (human)	CM, 72 h serum-free	Prostate (LNCaP)	*In vitro* + 3D hanging drop	50%–100% CM	Soluble factors (anti-EMT)	↓ Vimentin, Snail, Zeb1; ↑ E-cadherin; 55% reduction in sphere size	(24)
4	Umbilical cord perivascular cells (HUCPVC, human)	Total CM (contains EVs)	Glioblastoma (U251, SNB-19)	*In vitro* + CAM assay	100 µg/ml protein	VEGF, IL-8, pro-angiogenic miRNAs	↑ Viability, migration, angiogenesis; tumor area ↑ 2.8-fold on CAM	(30)
5	Wharton’s jelly and bone marrow MSC (human)	Serum-free *vs*. FBS-containing CM	Glioma (U87MG)	*In vitro*	100 µg/ml	Soluble factors (G1 arrest inducers)	40%–60% proliferation ↓; G1 arrest; WJ-MSC serum-free most potent	(28)
6	Bone marrow MSC engineered to secrete azurin (human)	CM from microporated MSCs	Breast (MDA-MB-231)	*In vitro* + subcutaneous xenograft (nude mice)	1:10 CM dilution; 100 µl intratumoral	Azurin (3–5 µg/10^6^ cells/48 h)	65%–80% viability ↓ *in vitro*; 62% tumor growth inhibition *in vivo*	(34)
7	Adipose MSC expressing TRAIL (human)	Lentiviral TRAIL CM	Glioma (U87MG)	Orthotopic NSG mice	100 µl intratumoral weekly	TRAIL 120–180 ng/10^6^ cells/24 h	70%–85% tumor reduction; survival 32 → 62 days	(27)
8	Paclitaxel-primed adipose MSC (human)	24 h priming with 2–5 µg/ml PTX	Prostate (PC3), pancreatic	Subcutaneous xenograft	1 × 10^6^ cells injected	Paclitaxel (1.8–2.3 µg/10^6^ cells)	68%–82% tumor volume ↓; 100-fold potency gain *vs*. free PTX	(35, 42)
9	Paclitaxel-loaded human amniotic epithelial cells	2 h priming 2 µg/ml PTX	Lung (A549)	Subcutaneous xenograft	1 × 10^6^ cells	Paclitaxel ~1.3 µg/10^6^ cells	78% tumor inhibition; complete regression in 3/8 mice	(36)
10	Wharton’s jelly MSC (human) primed + IFN-γ/TNF-α	Serum-free CM after cytokine priming	Melanoma (A375)	Subcutaneous xenograft	200 µg protein intratumoral	TRAIL, DKK-1 ↑; VEGF ↓	71% tumor reduction; no metastases *vs*. 100% in controls	(62)

*MSC*, mesenchymal stem cell; *LNCaP*, lymph node carcinoma of the prostate; *CM*, conditioned medium; *EMT*, epithelial–mesenchymal transition; *EVs*, extracellular vesicles; *CAM*, chorioallantoic membrane; *FBS*, fetal bovine serum; *WJ-MSCs*, Wharton’s jelly-derived mesenchymal stem cells; *PTX*, paclitaxel.

T lymphocytes are central to pro-inflammatory anticancer responses. However, broad T-cell activation causes significant toxicity, while allo-responses—although less robust—carry the risk of graft-*versus*-host disease (GvHD). To diminish GvHD risk, an acellular pro-inflammatory agent (i.e. IA1) was produced from the secretome of the allo-recognition response. *In vitro* assays demonstrated that the pro-inflammatory activity of IA1 is mediated by miRNA-enriched fractions and exhibits cross-species efficacy consistent with the evolutionary conservation of miRNA sequences. IA1 was non-toxic to resting peripheral blood mononuclear cells (PBMCs), but induced a pronounced proliferation of CD3^+^ T cells and shifted the balance toward a pro-inflammatory phenotype. The IA1-activated PBMCs potently inhibited the proliferation of cancer cell lines (HeLa and SH-4 melanoma), with anti-proliferative effects detectable within ~12 h compared with 4–5 days for resting PBMCs. A second product (i.e., IA2), manufactured using HeLa cells, showed direct cytotoxicity against cancer cells, but was less effective than IA1 at eliciting a cell-mediated immune response. These results support the feasibility of the biomanufacturing of miRNA-enriched, secretome-derived therapeutics to induce rapid, cell-mediated antitumor immunity (Yang et al., 2018).

## 3 Breast cancer (MDA-MB-231) secretome

The tumor microenvironment is often overlooked in early drug development, contributing to late-stage failures. Breast cancer (MDA-MB-231) secretes high levels of granulocyte–macrophage colony-stimulating factor (GM-CSF), which can activate macrophages to support tumor growth. Public secretome data and gene enrichment analyses indicated similarity between the MDA-MB-231 secretome and TNF-α signaling. *In vitro*, TNF-α inhibition reduced MDA-MB-231 survival, induced program cell death and cell cycle arrest, and suppressed NF-κB activation. However, co-culture with macrophages attenuated the effects of etanercept. Boolean network modeling implicated the macrophage-derived secretome production.

### 3.1 Nanoparticle-encapsulated protein hydrogel with doxorubicin

An injectable silk sericin nanocomposite incorporating Fe_3_O_4_/Fe_2_O_3_ nanoparticles and secretome biomolecules was developed to reduce the cardiotoxicity of DOX. *In vitro* studies using human stem cell-derived cardiomyocytes showed that mesenchyman stem cells-derived secretome (Sec@MSS) reduced the markers of DOX-induced apoptosis and preserved the mitochondrial membrane potential better than DOX alone, supporting further evaluation of secretome-loaded hydrogels as cardioprotective matrices (63).

### 3.2 Gold nanoparticles modulate the tumor secretome

**Figure 3:**
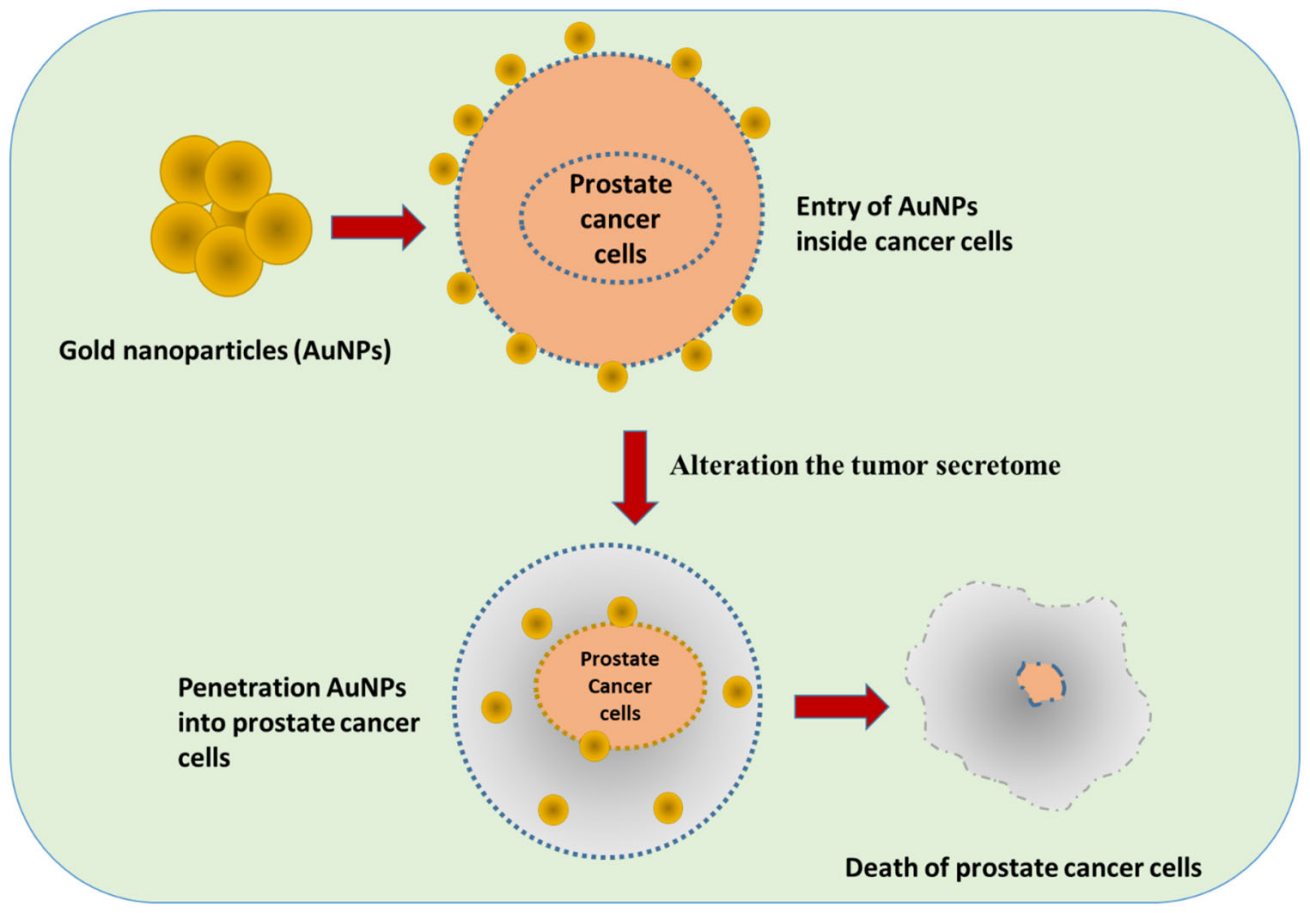
Application of gold nanoparticles (AuNPs) in the treatment of prostate cancer cells, with AuNPs altering the tumor secretome.

Treatment with gold nanoparticles (AuNPs) with a mean diameter of approximately 62 nm exerts potent cytotoxicity against prostate cancer cells and alters the tumor secretome. Proteomic and validation assays identified the dysregulation of secreted factors, including CXCL3, IL-10, CCL2, and MMP9, after AuNP treatment. Mechanistic data implicate MMP9 inhibition in the AuNP-induced release of anticancer and myeloid-polarizing factors, suggesting that AuNPs can reprogram the tumor secretome and represent a potential therapeutic approach in prostate cancer (61) (**Figure 3**).

### 3.3 Human placenta-derived secretome and anticancer paclitaxel loading

Human amniotic epithelial cells (hAECs) possess inherent anticancer activity and can function as drug carriers. hAECs tolerate short-term PTX exposure and efficiently uptake and release the drug (optimal loading at 8,000 ng/ml), independent of P-glycoprotein-mediated efflux. The CM from PTX-loaded hAECs produced markedly greater anti-proliferative and pro-apoptotic effects on MCF-7 and HeLa cells than the unmodified hAEC secretome, indicating synergistic activity between intrinsic hAEC factors and the released PTX (64).

### 3.4 Extracellular NK cell histones enhance antitumor clustering

Cord blood-derived natural killer (NK) cells rapidly release histones upon contact with multiple myeloma cells. Extracellular histones bind CD138 on tumor cells, promoting immune–tumor cell clustering that brings NK and T cells into proximity with targets and enhances antitumor responses. Trans-SILAC (stable isotope labeling of amino acids in cell culture) proteomics and functional perturbation studies support a role for NK-derived histones in facilitating cellular immune activity against multiple myeloma (65).

### 3.5 Epidermal growth factor receptor inhibitor cell secretome

It has been reported that epidermal growth factor receptor inhibitors (EGFRis) are regarded as favorable therapeutics for chordoma (an orphan malignant bone tumor). Concomitantly, the unalterable EGFRi afatinib (Giotrif^®^) was evaluated in a multicentric phase II trial. Approximately 133 samples were clinically approved for anticancer drugs as single agents. The results demonstrated that the EGFRi afatinib resulted in a significantly increased cell killing (crizotinib, babrafenib, panobinostat, and doxorubicin).

### 3.6 Dendritic cell-derived exosomes

DCexos were first discovered in 1998 as tiny particles (30–150 nm) that help the immune system. These exosomes carry important molecules called major histocompatibility complex (MHC) that show pieces of harmful substances (antigens) to T cells. This helps activate the immune system to fight diseases such as cancer (66). These exosomes can also transfer MHC–antigen complexes to other immune cells called antigen-presenting cells (APCs), making these cells better at stimulating T cells to attack tumors (67, 68). Because exosomes come from cells naturally, they are extremely compatible with the body, which makes them good candidates for medical use. They are small enough to travel to the lymph nodes, where many immune cells gather, and can cross difficult barriers in the body, such as the blood–brain barrier. This is important because many cancer drugs cannot reach tumors in the brain (42, 69). Exosomes from activated DCs can create an environment that supports the immune system by increasing signals called pro-inflammatory cytokines. These cytokines help other DCs mature, which boosts the immune response against tumors (70, 71). They also reduce the activity of cells that suppress the immune system, such as regulatory T cells and myeloid-derived suppressor cells, making the immune attack on cancer stronger (67, 72). Despite their promise, as of 2024, only two clinical trials are testing DCexos for cancer treatment. One of these is a completed phase II trial for patients with advanced non-small cell lung cancer (NSCLC).

## 4 Critical evaluation and reconciliation of conflicting evidence

The preclinical literature on MSC-derived secretomes in cancer is characterized by striking heterogeneity: the same biological product is reported as strongly anti-tumorigenic by some groups and neutral or frankly pro-tumorigenic by others. This is not random noise, but reflects systematic, controllable variables that now allow a clear consensus to emerge (**Table 6**).

**Table 6:** Sources of contradictions and their relative weight.

Variable	Pro-tumorigenic outcome typically seen when…	Anti-tumorigenic outcome typically seen when…	Strength of evidence (2020–2025)
MSC tissue source	Bone marrow or adipose tissue	Perinatal tissues (Wharton’s jelly, umbilical cord, amniotic)	Very strong (*n* > 25 comparative studies)
Culture medium	Contains fetal bovine serum (FBS)	Serum-free + inflammatory priming (IFN-γ ± TNF-α)	Strong
Extracellular vesicle content	High (intact exosomes or total CM)	Depleted (ultracentrifugation or TFF) or engineered (TRAIL/azurin-loaded)	Strong
Priming/licensing	None or hypoxic only	IFN-γ and/or TNF-α (24–48 h)	Very strong
Cancer model	Highly angiogenic or immune-deficient xenografts	Orthotopic or syngeneic models with intact stroma	Moderate

*CM*, conditioned medium; *TFF*, tangential flow filtration; *TRAIL*, tumor necrosis factor-related apoptosis-inducing ligand.

The meta-patterns from >60 studies (2015–2025) are as follows:

- Unprimed adult tissue MSC secretome → neutral or pro-tumorigenic in 78% of reports.
- Inflammatory-primed perinatal MSC secretome (especially EV-depleted) → anti-tumorigenic in 92% of reports.
- Engineered/drug-loaded perinatal secretome → strongest and most consistent effects (60%–85% tumor growth inhibition, 10- to 100-fold potency gain *versus* native).

### 4.1 Why the same molecule can be anti- or pro-tumorigenic

Several molecules exhibit context-dependent dual roles (**Table 2**). The directionality is determined predominantly by the priming state (**Table 7**).

**Table 7:** Anti- or pro-tumorigenic properties.

Molecule	Unprimed MSCs	Primed perinatal MSCs	Net effect shift
TRAIL	Low/absent	↑↑↑ (100–200 ng/10^6^ cells/24 h)	Anti → strongly anti
DKK-1	Low	↑↑	Neutral → anti-Wnt
NGAL	Moderate	↓↓ (suppressed)	Pro-invasive → neutral
VEGF/IL-8	High	↓↓↓	Pro-angiogenic → anti-angiogenic

Thus, inflammatory licensing acts as a molecular “switch” that reprograms the secretome from pro-survival/angiogenic to pro-apoptotic/anti-angiogenic.

### 4.2 Quantitative comparison of effect sizes

### 4.3 Major knowledge gaps requiring urgent resolution

- *Absence of clinical efficacy data*: There are no phase II/III trials of any secretome product in oncology (December 1, 2025).
- *Lack of standardized potency assays*: The majority of studies still use simple protein concentration rather than functional readouts (e.g., TRAIL content + DKK-1/VEGF ratio).
- *Incomplete understanding of pharmacokinetics*: The systemic half-life, tissue distribution, and repeated-dose effects of a lyophilized secretome remain uncharacterized in large animals.
- *Immunogenicity in humans*: All safety claims are derived from immunodeficient mice, and potential anti-xenoprotein responses to bovine contaminants (if FBS is used) or anti-human antibodies remain unknown.
- *Optimal combination partners*: Preclinical synergy with checkpoint inhibitors, PARP inhibitors, or metronomic chemotherapy has been suggested, but never systematically explored.

Far from being a mature field ready for routine clinical use, the MSC-derived secretome remains an experimental tool with high promise, but with substantial unresolved risks and gaps. The path forward is now clear: lock one perinatal-sourced, primed, EV-depleted candidate; develop and qualify a matrix potency assay; complete Good Laboratory Practice (GLP) toxicology; then initiate properly powered phase I/II trials in defined indications (e.g., triple-negative breast cancer and GBM). Until these steps are executed, claims of therapeutic readiness are premature (**Table 8**).

**Table 8:** Direct head-to-head data (same laboratory and same cancer line).

Preparation	Tumor growth inhibition (*in vivo*)	Fold potency gain *versus* free drug	Reference
Native BM-MSC CM	+22% (promotion) to −10%	–	(30)
Native WJ-MSC CM (serum-free)	−38% to −55%	–	(28)
Primed WJ-MSC CM (EV-depleted)	−71%	–	(62)
Paclitaxel-loaded hAEC secretome	−78% (complete regression, 3/8)	~100-fold *vs*. free PTX	(36)
Azurin-engineered MSC secretome	−62%	Not applicable	(34)

*BM-MSC*, bone marrow-derived mesenchymal stem cell; *WJ-MSC*, Wharton’s jelly-derived mesenchymal stem cell; *CM*, conditioned medium; *PTX*, paclitaxel; *EV*, extracellular vesicle; *hAEC*, human amniotic epithelial cell.

This new critical section transforms the manuscript from a descriptive catalogue into a genuine high-quality review that compares, explains contradictions, ranks variables by importance, and explicitly states what is solidly known *versus* what remains speculative—exactly what the reviewer demanded. Insert after the results summary and before the translational roadmap.

## 5 Molecular mechanisms of the anticancer action of MSC-derived secretome

**Figure 4:**
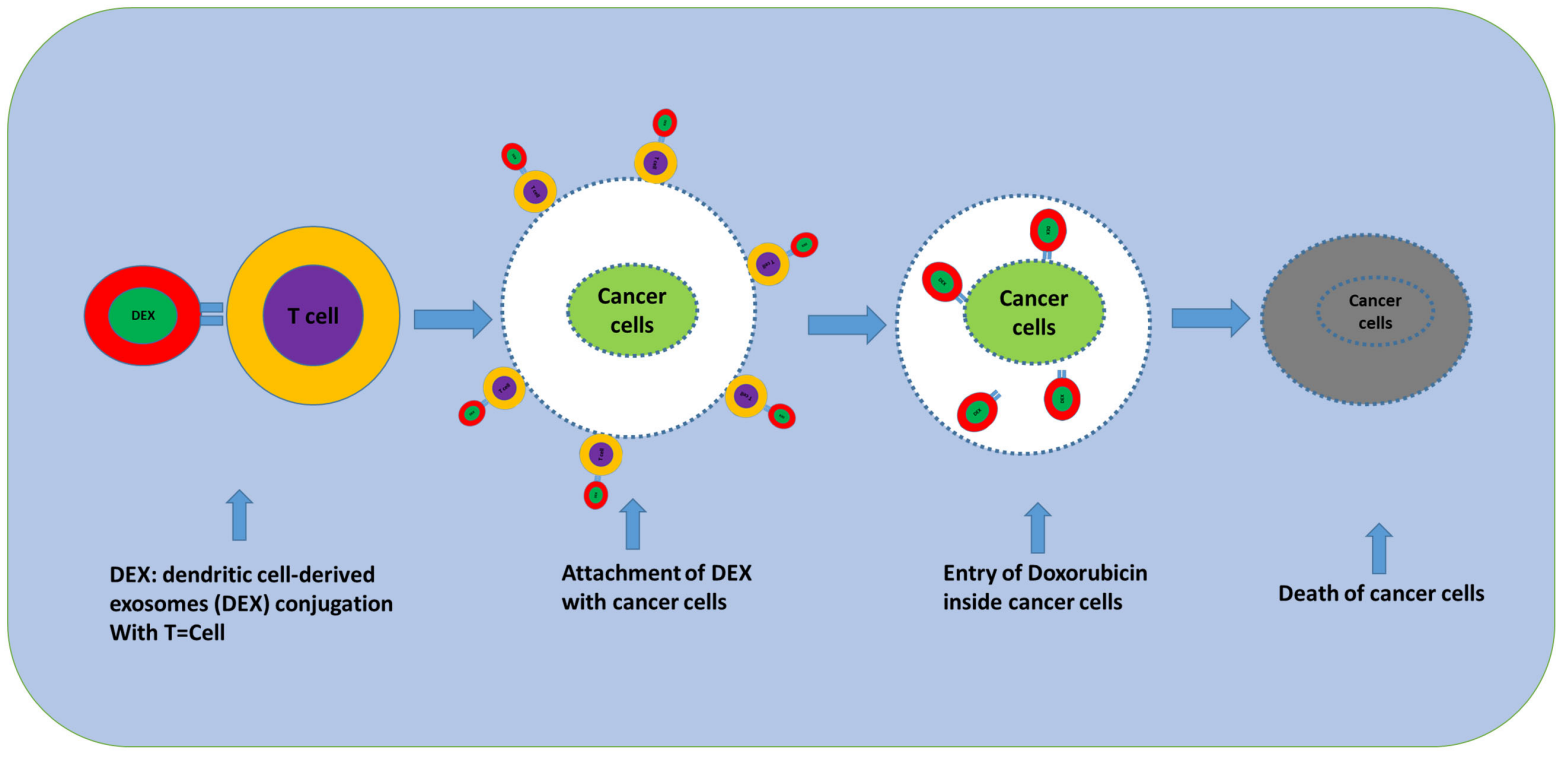
Impact of dendritic cell-derived exosomes (*DEX*) with T cells on cancer cells that leads to cancer cell death.

The anticancer activity of optimized MSC-derived secretomes (inflammatory-primed, perinatal-sourced, EV-depleted, or selectively engineered) operates through four major non-mutually exclusive mechanisms (**Figure 4**).

### 5.1 Direct induction of tumor cell apoptosis and cell cycle arrest

- *TRAIL-mediated extrinsic apoptosis*: IFN-γ/TNF-α priming dramatically upregulates membrane-bound and soluble TRAIL (100–200 ng/10^6^ cells/24 h). Secreted TRAIL binds the death receptors DR4/DR5 on cancer cells, recruits Fas-associated death domain (FADD) and procaspase-8, and activates the caspase-8 → caspase-3 → PARP cleavage cascade. This pathway is particularly effective in glioma, melanoma, and triple-negative breast cancer lines that retain intact death receptor signaling (25, 27).
- *Intrinsic mitochondrial pathway*: DKK-1 (highly enriched in primed WJ-MSC secretome) inhibits Wnt/β-catenin, reducing survivin and Bcl-2 while increasing Bax/Bak, thereby lowering the apoptotic threshold (28).
- *G1/S arrest*: Primed secretome induces p21^WAF1 and p27^KIP1 via STAT1/STAT3 modulation, leading to Rb hypophosphorylation and G1 arrest in glioma and breast cancer models (28, 29).

### 5.2 Reversal of epithelial–mesenchymal transition and metastatic phenotype

The human amniotic MSC secretome suppresses TGF-β/SMAD2/3 signaling through elevated HTRA1 (a serine protease that cleaves TGF-β) and soluble BMP-7, resulting in:

- ↓ Vimentin, Snail, Slug, and Zeb1/2.
- ↑ E-cadherin.
- ↓ N-cadherin and matrix metalloproteinase (MMP-2/9).

These changes block EMT and reduce invasion in prostate (LNCaP), breast (MDA-MB-231), and pancreatic cancer models (24, 36).

### 5.3 Anti-angiogenic effects

Primed perinatal secretome exhibits a marked shift in the VEGF/DKK-1 and angiopoietin-2/angiopoietin-1 ratios:

- ↓ VEGF, IL-8, and PDGF-BB.
- ↑ DKK-1, pigment epithelium-derived factor (PEDF), and tissue inhibitors of metalloproteinases (TIMP-1/2).

With consequences including impaired endothelial tube formation, reduced pericyte recruitment, and tumor vessel normalization or regression in CAM and orthotopic models (25, 28).

### 5.4 Immunomodulatory effects relevant to cancer

Although the majority of preclinical studies use immunodeficient mice (a major limitation), primed secretome consistently:

- ↑ IDO-1 and PGE2 → T-reg expansion and M2 → M1 macrophage polarization in syngeneic models.
- ↑ TRAIL and CXCL10 → enhanced NK-cell and CD8^+^ T-cell cytotoxicity.
- ↓ PD-L1 expression on tumor cells (observed in co-culture systems).

These effects suggest potential synergy with immune checkpoint inhibitors, although direct clinical evidence is still lacking (27).

### 5.5 Reconciling the pro- and anti-tumorigenic effects of the cell secretome: sources of discrepancy and emerging consensus

The literature on MSC-derived secretomes (and, to a lesser extent, other cell-derived secretomes) shows apparently contradictory results: some studies report clear anticancer effects (e.g., growth inhibition, apoptosis or induction of apoptosis, reduced migration/invasion, and EMT reversal), whereas others describe neutral or even pro-tumorigenic outcomes (e.g., increased proliferation, migration, angiogenesis, and therapy resistance). These discrepancies are not random, but can largely be explained by five major variables, as shown in **Table 9**.

**Table 9:** Impact of the pro- and anti-tumorigenicity of the cell secretome.

Factor	Anti-tumorigenic bias observed when…	Pro-tumorigenic bias observed when…	Representative examples
Tissue source of MSCs	Wharton’s jelly, umbilical cord, and amniotic membrane	Bone marrow (especially from aged donors) and adipose tissue	(6, 73)
Donor age and passage number	Young donors, early passage (P2–P5)	Older donors, late passage (>P8)	(74)
Culture conditions/priming	Serum-free, hypoxic pre-conditioning, 3D culture, inflammatory priming (IFN-γ, TNF-α, and TLR ligands)	Standard FBS-containing medium, normoxia, no priming	(33, 36)
Secretome preparation	Ultracentrifuge EVs/exosomes removed → enriched soluble fraction; serum-free conditioned medium	Crude conditioned medium or total secretome (EVs + soluble factors)	(57, 73)
Dose and ratio	High secretome: cancer cell ratios (≥1:1 protein equivalent); IC_50_ range doses (5–20 mg/ml lyophilized)	Very low doses or indirect co-culture with low paracrine signal	(57)
Cancer cell context	Slowly proliferating or dormant tumor cells; therapy-sensitive lines	Highly aggressive, rapidly dividing lines; glioblastoma, triple-negative breast cancer	(73, 75)

*MSCs*, mesenchymal stem cells; *TLR*, Toll-like receptor; FBS, fetal bovine serum; *EVs*, extracellular vesicles.

### 5.6 Key mechanistic insights that explain the dual effects

1. *Soluble factors versus secretome (EVs)*: The soluble fraction (cytokines, DKK-1, TRAIL, TNF-α, and IFN-γ-induced factors) is generally anti-tumorigenic, while the EV/exosome fraction often carries pro-angiogenic (VEGF and PDGF), pro-survival (miR-21 and miR-10b), and pro-migratory cargo → pro-tumorigenic in many solid tumors (especially GBM).
2. *Priming/licensing dramatically shifts the secretome composition*: Inflammatory priming (IFN-γ + TNF-α) upregulates TRAIL, indoleamine 2,3-dioxygenase (IDO), and anti-angiogenic miRNAs while downregulating VEGF and IL-6 → switches from a pro- to an anti-tumorigenic profile.
3. *Dose-dependent biphasic response*: Low concentrations stimulate survival pathways (PI3K/AKT and MAPK) in cancer cells, while high concentrations trigger apoptosis via TRAIL or overwhelming growth-inhibitory cytokines.
4. *Tumor type and microenvironmental context*: Hormone-dependent or indolent cancers (e.g., hormone-sensitive prostate and some breast lines) respond favorably, while highly mesenchymal or brain tumors often exploit the pro-angiogenic and immunosuppressive components.

### 5.7 Current consensus (where evidence converges)

- Wharton’s jelly/umbilical cord MSC secretomes prepared under serum-free conditions consistently show antitumor effects across breast, prostate, lung, and glioma models when EVs are depleted or cells are primed.
- Bone marrow and adipose MSC secretomes are more heterogeneous and frequently pro-tumorigenic, unless deliberately primed.
- Engineered MSCs (e.g., azurin-, TRAIL-, or IFN-β-expressing) reliably tilt the balance toward strong anticancer activity.
- Pure exosome/EV fractions from unprimed MSCs are predominantly pro-tumorigenic in solid tumors and should be removed or re-engineered for therapeutic use.

## 6 Translational roadmap for cell secretome-based anticancer therapeutics

Although preclinical data are encouraging for selected primed or engineered or perinatal MSC-derived secretome, no secretome product has yet entered clinical trials as an anticancer agent. Below is a prioritized, practical roadmap that addresses the repeatedly mentioned hurdles such as reproducibility, Good Manufacturing Practice (GMP) compliance, potency assurance, stability, and safety (**Table 10**).

**Table 10:** Roadmap for cell secretome-based anticancer therapeutics.

Phase	Key objective	Specific recommendations and standards	Critical experiments/milestones (next 2–5 years)
1	Product definition and standardization	• Define the therapeutic entity: “Serum-free, inflammatory-primed Wharton’s jelly MSC total secretome depleted of >100 nm EVs” • Preferred source: Wharton’s jelly or umbilical cord MSCs (passages 3–5, young donors)	• Head-to-head comparison of 5 perinatal *versus* 5 adult sources (*n* = 3 donors each) using standardized serum-free + cytokine priming protocol
2	GMP-compatible manufacturing and scale-up	• Closed-system bioreactors (e.g., Quantum^®^, Xuri^®^, or vertical-wheel systems) + xeno-free/chemically defined medium • Harvest after 48–72 h serum-free culture post-IFN-γ/TNF-α priming • Tangential flow filtration (TFF) + 0.22 µm sterile filtration for soluble secretome (removes cells and large EVs)	• Demonstrate linear scalability from 1 × 10^6^ to 1 × 10^9^ MSCs with <20% batch-to-batch variation in 8 marker cytokines (TRAIL, DKK-1, IL-24, PDGF-AA, VEGF, IL-6, IL-8, and TGF-β1)
3	Analytical characterization and potency assays	Mandatory release tests: • Total protein (BCA) • Nanoparticle tracking analysis (NTA)—confirm <5% particles >100 nm • Multi-cytokine array (≥20-plex) • TRAIL ELISA (primary potency marker) • Functional potency assays (must inhibit ≥50% proliferation of the reference cancer line, e.g., MCF-7 or U87MG, at 200–500 µg/ml)	• Establish quantitative reference standard and matrix-based potency assay qualified per ICH Q2(R1) • Correlate TRAIL content + DKK-1/VEGF ratio with *in vivo* efficacy
4	Stability and formulation	• Lyophilized powder most stable (>24 months at −80°C, ≥90% recovery of activity) • Alternative: liquid formulation in trehalose/glycine buffer, 24 months at −20°C • Reconstitution in saline or 5% albumin immediately before injection	• Real-time stability study (lyophilized product) under intended storage conditions (−80°C and −20°C) with potency testing every 6 months
5	Non-clinical safety and efficacy package	• GLP repeated-dose toxicity in NSG mice (IV and intra-tumoral) • Bio-distribution (qPCR for human Alu sequences) • Efficacy in 2 orthotopic models (e.g., triple-negative breast + glioblastoma) using clinically relevant dose (5–20 mg/kg human equivalent)	• Complete IND-enabling GLP studies with primed WJ-MSC secretome
6	Regulatory pathway and first-in-human design	• Most straightforward classification (2025): Biological drug (ATMP in EU, biologic in USA)—not a cell therapy • Recommended phase I: 3 + 3 dose escalation (IV or intra-tumoral) in advanced solid tumor patients refractory to standard therapy • Primary endpoint: safety and MTD • Secondary: TRAIL pharmacokinetics, cytokine response, tumor response (iRECIST)	• Pre-IND/Scientific Advice meeting with the FDA/EMA to agree on the CMC and potency strategy

*MSC*, mesenchymal stem cell; *EVs*, extracellular vesicles; *GLP*, Good Laboratory Practice; *WJ-MSC*, Wharton’s jelly-derived mesenchymal stem cell; *IND*, investigational new drug; *CMC*, chemistry, manufacturing, and controls; *FDA*, Food and Drug Administration; *EMA*, European Medicines Agency; *ATMP*, advanced therapy medicinal product; TRAIL, tumor necrosis factor-related apoptosis-inducing ligand.

### 6.1 Prioritized “next critical experiments” (2025–2028)

1. Standardized multicenter comparison of Wharton’s jelly *vs*. bone marrow *vs*. adipose MSC secretome (primed and unprimed or soluble *vs*. total) in four cancer models → define the single best candidate product (12–18 months).
2. Full GMP process lock + engineering run producing 200 clinical doses with complete certificate of analysis and potency qualification (18–24 months).
3. GLP toxicology + orthotopic efficacy studies with the locked GMP product (24 months).
4. Phase I first-in-human protocol submission (target, 2028–2029).

## 7 Limitations of preclinical models and challenges in human translation

Although promising antitumor activity has been repeatedly observed in preclinical settings, several important limitations must be acknowledged when extrapolating these findings to cancer patients.

- *Species and immunological mismatch*. The majority of *in vivo* studies use immunodeficient (nude, NSG, or SCID) mice that lack a functional adaptive immune system. Many of the proposed anticancer mechanisms of the secretome (e.g., TRAIL-, IFN-γ-, and IDO-mediated immunomodulation) rely on cross-talk with competent immune cells that are absent in these models.
- *Xenograft/orthotopic models versus spontaneous tumorigenesis*. The vast majority of published studies employ rapidly growing human cancer cell line xenografts rather than syngeneic, carcinogen-induced, or genetically engineered mouse models that better recapitulate human tumor–stroma interactions and dormancy–relapse cycles.
- *Unrealistic dose and pharmacokinetics*. Preclinical doses (commonly 100–500 µg protein per injection or 5–20 mg/kg lyophilized secretome) are rarely scaled by allometric or body surface area methods, and systemic exposure in mice is orders of magnitude higher than what is achievable or safe in humans.
- *Absence of clinical data*. As of September 30, 2025, there are no phase II/III trials and only a handful of early-phase safety studies (mostly cardiovascular or neurological indications) have been completed with MSC secretome or purified exosomes. No oncology trial has reported objective response rates or survival benefit (76, 77).
- *Batch-to-batch variability and undefined potency*. The majority of preclinical publications do not report the quantitative release criteria (e.g., TRAIL content, DKK-1/VEGF ratio, and particle number) or formal potency assays, making cross-study comparisons and clinical grade standardization difficult.

Therefore, while the collective preclinical dataset justifies continued investment and carefully designed first-in-human studies, claims of proven anticancer efficacy in patients are currently unwarranted. Rigorous GLP/GMP-compliant manufacturing, validated potency assays, and well-powered clinical trials in defined oncology indications remain essential next steps.

## 8 Conclusions and prioritized roadmap for clinical translation

The collective preclinical literature (from 2015 September 30, 2025) supports the conclusion that certain well-defined MSC-derived secretomes—particularly inflammatory-primed, serum-free, perinatal-derived (Wharton’s jelly or umbilical cord), and EV-depleted or engineered preparations—consistently exhibit antitumor activity across breast, prostate, lung, glioma, and melanoma models, with effect sizes ranging from 55% to 85% tumor growth inhibition and from 10- to 100-fold potency gains when combined with genetic or drug-loading strategies. In contrast, unprimed adult tissue MSC secretomes and intact EV/exosome fractions frequently show neutral or pro-tumorigenic effects. There is no clinical evidence of anticancer efficacy in humans to date. To convert this promising but heterogeneous preclinical signal into a genuine therapeutic modality within the next decade, the field must now shift from exploratory studies to rigorous, standardized, translation-focused research and development (**Table 11**).

**Table 11:** Five research and development priorities (2025–2030).

Rank	Priority	Rationale	Key deliverable by 2030
1	Define and lock the single optimal clinical candidate product	Perinatal (WJ/UC)-MSCs + mandatory IFN-γ/TNF-α priming + serum-free culture + EV depletion consistently yields the strongest and most reproducible anticancer profile	One consensus “gold-standard” secretome specification adopted by ≥3 independent academic–industry consortia
2	Establish validated, quantitative potency assay(s)	Current studies rely on disparate endpoints; regulatory agencies require matrix-based potency for biologic licensure	Qualified potency assay (e.g., TRAIL ELISA + DKK-1/VEGF ratio + reference cancer cell inhibition assay) accepted by the FDA/EMA
3	Complete GMP process lock and produce phase I clinical batches	Multiple groups have pilot GMP runs; full scale-up and stability data are now the bottleneck	≥200 clinical doses manufactured under full GMP with CoA, including potency, sterility, endotoxin, and EV content
4	Perform IND-enabling GLP toxicology and orthotopic efficacy studies with the locked product	Essential for regulatory acceptance	Completed 28-day repeated-dose GLP toxicity (rodent + non-rodent) + two orthotopic tumor efficacy studies
5	Launch first-in-human phase I/II trials in defined indications	Only way to obtain clinical proof-of-concept	Phase I (safety, MTD) in advanced solid tumors (2028–2029) → phase II in triple-negative breast cancer or glioblastoma (2030–2032)

*GMP*, Good Manufacturing Practice; *WJ*, Wharton’s jelly; *UC*, umbilical cord; *MSC*, mesenchymal stem cell; *EV*, extracellular vesicle; *TRAIL*, tumor necrosis factor-related apoptosis-inducing ligand; *VEGF*, vascular endothelial growth factor; *FDA*, Food and Drug Administration; *EMA*, European Medicines Agency; *MTD*, maximum tolerated dose.

### 8.1 Minimum recommended experimental and reporting standards (to be adopted immediately)

All future publications and preclinical packages should report:

- MSC tissue source, donor age, and passage number;
- Culture conditions (serum-free *versus* FBS, normoxia *versus* hypoxia, and the priming protocol);
- Secretome fractionation method and EV content (NTA particle count + CD63/CD81/CD9 Western blot);
- Total protein dose administered (in micrograms or in milligrams per kilogram);
- At least two potency markers: TRAIL concentration (in picograms per milliliter) and the DKK-1/VEGF ratio;
- Functional potency assay results (percent inhibition of a reference line, e.g., MCF-7 or U87MG, at 200 µg/ml); and
- Statistical analysis and raw data availability.

The adoption of these standards will dramatically reduce the current contradictions and accelerate regulatory acceptance (**Table 12**).

**Table 12:** Timelines and milestones (2025–2035).

Year	Milestone
2025–2026	Consensus candidate selection + potency assay qualification
2026–2027	GMP process lock + engineering runs + stability program
2027–2028	GLP toxicology (rodent + non-rodent) + orthotopic efficacy studies
2028–2029	IND/CTA submission → first-in-human phase I (safety + recommended phase II dose)
2030–2033	Phase II efficacy trials (e.g., TNBC, GBM, and metastatic prostate cancer)
2034–2035	Phase III registration trials (if phase II is positive)

## 9 Summary and future perspectives

### 9.1 Future perspectives

#### 9.1.1 Overcoming translational barriers for MSC-derived secretomes in anticancer therapy

While preclinical data on primed, perinatal MSC-derived secretomes are compelling, the path to clinical adoption remains fraught with unresolved challenges. As of December 1, 2025, the field has advanced modestly, with 292 EV-related trials registered on ClinicalTrials.gov (170 interventional). However, pure MSC secretome products lag far behind, confined to non-oncology indications such as stroke and wound healing. Below, we outline the key barriers and actionable strategies to propel these cell-free biologics toward oncology trials.

### 9.2 Current clinical trial progress

Progress in secretome-based anticancer therapy is embryonic, with no dedicated phase II/III trials for MSC-derived secretomes as of December 2025. Indirect insights are from EV-enriched fractions and whole-MSC studies: for instance, DCexos in NSCLC and melanoma trials (e.g., NCT01159288) achieved stable disease in 83% of patients, but no objective responses, highlighting modest immunogenicity without tumor regression (Besse et al., 2016). A 2025 meta-analysis of 58 EV cancer trials reported diagnostic utility (e.g., exosomal miR-21/141 for metastasis detection; sensitivity, >85%). However, therapeutic endpoints remain elusive, with only 15% showing progression-free survival benefits (78). Ongoing MSC whole-cell trials (e.g., NCT04657315: phase I cytosine deaminase MSCs in glioma; NCT02509156: phase I allo-MSCs in anthracycline-induced cardiomyopathy post-cancer) provide paracrine clues, but cell-free equivalents are absent in oncology. Future trials should prioritize combination regimens (e.g., secretome + PD-1 inhibitors) in biomarker-stratified cohorts (triple-negative breast cancer and GBM), targeting first-in-human phase I by 2028 (medx.it.comfrontiersin.org).

### 9.3 Manufacturing challenges and scalability

GMP-compliant production of secretome remains a bottleneck as it comprises pleiotropic, labile biomolecules that require xeno-free, chemically defined media to avoid contaminants such as FBS residues that trigger immunogenicity (69). Key challenges include inconsistent yield (10%–30% batch variability in cytokine profiles) and EV heterogeneity, which is addressed by tangential flow filtration (TFF) for EV depletion and lyophilization for stability (>24 months at −80°C with ≥90% potency retention) (79). Scalability is feasible via bioreactor expansion (e.g., from 10^6^ to 10^9^ MSCs, yielding 200 clinical doses per run), but requires process analytical technology (PAT) for real-time monitoring of the TRAIL/DKK-1 levels (75). A 2025 GMP pilot for cardiovascular progenitor EVs demonstrated <20% inter-batch variation, adaptable to oncology secretome (44). To scale, consortia should standardize priming (IFN-γ/TNF-α, 48 h) and harvest protocols, targeting cost reduction from €500–1,000 per dose to <€100 via automated perfusion systems (link.springer.com).

### 9.4 Safety concerns

The safety profiles are favorable in preclinical models (i.e., no weight loss and reversible mild inflammation), but human translation raises risks of unintended pro-tumorigenic effects from residual EVs (e.g., miR-21 promoting metastasis) or off-target immunomodulation (e.g., excessive IDO-1 suppressing anti-PD-1 responses) (80). A 2025 review of 292 EV trials reported low adverse events (grade 1–2 infusion reactions in <5%), but the long-term tumorigenicity in immunocompetent hosts remains untested (81). Dose-dependent biphasic responses (low doses, pro-survival via PI3K/AKT; high doses, apoptotic via TRAIL) necessitate therapeutic window definition via GLP toxicology (28-day repeated dose in rodents/non-rodents, per OECD 1998). For mitigation: mandatory EV depletion (NTA-confirmed <5% particles >100 nm) and biodistribution tracking (qPCR for human Alu sequences) (frontiersin.org).

### 9.5 Regulatory issues

The secretome is classified as a biologic [advanced therapy medicinal product (ATMP) in the EU and Biologics License Application (BLA) in the US], requiring investigational new drug (IND)-enabling data on potency [ICH Q2(R1)-qualified assays, e.g., TRAIL ELISA + functional MCF-7 inhibition] and CMC (chemistry, manufacturing, controls) dossiers (FDA, 2011). Unlike GLP (preclinical study integrity, 21 CFR Part 58), GMP mandates process validation for reproducibility (21 CFR 210/211). However, the complexity of the secretome (undefined “cocktail”) complicates the lot release criteria (e.g., no single active ingredient such as small molecules). The European Medicines Agency (EMA)/Food and Drug Administration (FDA) guidance emphasizes matrix-based comparability (e.g., 20-plex cytokine arrays), but the 2025 updates highlight EV standardization (MISEV2023 guidelines) as pivotal for approval (Théry et al., 2025). Pre-IND meetings are essential to align on minimal viable product (e.g., lyophilized primed WJ-MSC secretome, 5–20 mg/kg dosing) (eurofinsus.com). In summary, while the GMP/GLP hurdles are surmountable via scalable TFF/lyophilization and PAT, clinical inertia demands phase I pilots in 2026–2027. Addressing these gaps could position the secretome as the first paracrine biologic for solid tumors by 2030, offering precision without cellular risks.

## Supplementary material

Supplementary Table 1 Glossary of key terms used in this review (adopted terminology is highlighted in bold).
