# Supplementary material for: Cell secretome as a potential anticancer therapeutic agent: composition, mechanisms, preclinical evidence, and translational challenges
Source: Front Oncol. 2026 Mar 4;16:1729022. doi: 10.3389/fonc.2026.1729022 (PMC12995652; doi:10.3389/fonc.2026.1729022)
Supplement: Supplementary Table 1 — Glossary of key terms used in this review (adopted terminology is highlighted in bold). [file Table1.docx]

**Table-S1. Glossary of key terms used in this review (adopted terminology is highlighted in bold)**

| **Term** | **Definition used in this review** | **Synonyms used in the literature (avoided here)** |
| --- | --- | --- |
| Secretome | The entire set of molecules (soluble factors + extracellular vesicles) secreted by a cell/tissue/organism into the extracellular space at a given time and under defined conditions | Cell secretion, cell secretory products |
| MSC-derived secretome | Secretome produced by mesenchymal stem/stromal cells (MSCs) regardless of tissue origin | MSC secretome, MSC-conditioned medium (CM) |
| Conditioned medium (CM) | The complete culture supernatant harvested from cells (contains both soluble factors and extracellular vesicles unless further fractionated) | Crude secretome, total secretome |
| Soluble secretome fraction | Protein- and peptide-rich fraction after removal of extracellular vesicles (typically by 100 000–120 000 × g ultracentrifugation or 0.22 µm + TFF) | Secretome, soluble factors |
| Extracellular vehicles (EVs) | Heterogeneous population of membrane-bound particles (exosomes 30–150 nm, microvesicles 100–1000 nm, apoptotic bodies >1 µm) released from cells | Exosomes, micro vesicles, microparticles |
| Exosomes | Small EVs (30–150 nm) of endosomal origin, typically enriched by ultracentrifugation or polymer precipitation | — |
| Priming / licensing | Pre-treatment of MSCs with pro-inflammatory cytokines (IFN-γ ± TNF-α, TLR agonists, hypoxia) to shift the secretome toward anti-tumorigenic profile | Activation, pre-conditioning |
